# Prospective comparison on cardiac iron by MR in thalassemia major patients treated with combination deferipron-desferrioxamine versus deferipron and desferrioxamine in monoterapy

**DOI:** 10.1186/1532-429X-13-S1-O18

**Published:** 2011-02-02

**Authors:** Alessia Pepe, Antonella Meloni, Giuseppe Rossi, Maria Chiara Dell'Amico, Marcello Capra, Vincenzo Caruso, Lorella Pitrolo, Michele Centra, Pasquale Pepe, Eliana Cracolici, Paolo Ricchi, Massimo Lombardi

**Affiliations:** 1 “G. Monasterio” Foundation and Institute of Clinical Physiology, CNR, Pisa, Italy; 2 P.O. G. Di Cristina, ARNAS Civico, Palermo, Italy; 3 P.O. "S. Luigi-Currò" - ARNAS Garibaldi, Catania, Italy; 4 Az. Osp. "Villa Sofia", Palermo, Italy; 5 Servizio Trasfusionale OO.RR. Foggia, Foggia, Italy; 6 Policlinico "Paolo Giaccone", Palermo, Italy; 7 A.O.R.N. Cardarelli, Napoli, Italy

## Background

Combination therapy with deferipron and desferrioxamine (DFP+DFO) seems more effective than DFP and DFO in monotherapy in removing myocardial iron. However, no data are available in literature about prospective comparisons on cardiac iron and function in TM patients treated with DFP+DFO versus DFP and DFO in monotherapy. Aim: The aim of this multi-centre study was to assess prospectively in a large clinical setting the efficacy of the DFP+DFO versus DFP and DFO in TM patients by quantitative MR.

## Methods

Among the first 739 TM patients enrolled in the MIOT (Myocardial Iron Overload in Thalassemia) network, 253 patients performed a MR follow up study at 18±3 months according to the protocol. We evaluated prospectively the 43 patients treated with DFP+DFO versus the 30 patients treated with DFP and the 66 patients treated with DFO between the 2 MR scans. Myocardial iron concentrations were measured by T2* multislice multiecho technique. Biventricular function parameters were quantitatively evaluated by cine images.

## Results

Excellent/good levels of compliance were similar in the 3 groups (DFP+DFO 91% versus DFP 97% versus DFO 92%; P =0.76). Among the patients with no significant myocardial iron overload (MIO) at baseline (global heart T2*≥20 ms), there were no significant differences between groups to maintain the patients without MIO (DFP+DFO 90% versus DFP 100% versus DFO 100%; P=0.053). Among the patients with MIO at baseline (global heart T2*<20 ms), in all groups there was a significant improvement in the global heart T2* value (DFP+DFO P=0.0001, DFP P=0.001 and DFO P=0.003), in the number of segment with a normal T2* value (DFP+DFO P=0.0001, DFP P=0.031, DFO P=0.0001) and in the right global systolic function (DFP+DFO P=0.002, DFP P=0.031, DFO P=0.045). The improvement in the global heart T2* was significantly different among groups (mean difference global heart T2* DFP+DFO 6.6±6.5 ms, DFP 10.7±7.2, DFO 3.6±5.4; P=0.009). The improvement in the global heart T2* was significantly higher in the DFP+DFO versus the DFO group (P=0.017), but it was not significantly different in the DFP+DFO versus the DFP group (P=0.36) (see figure). The changes in the global systolic bi-ventricular function were not significantly different among groups.

**Figure 1:**
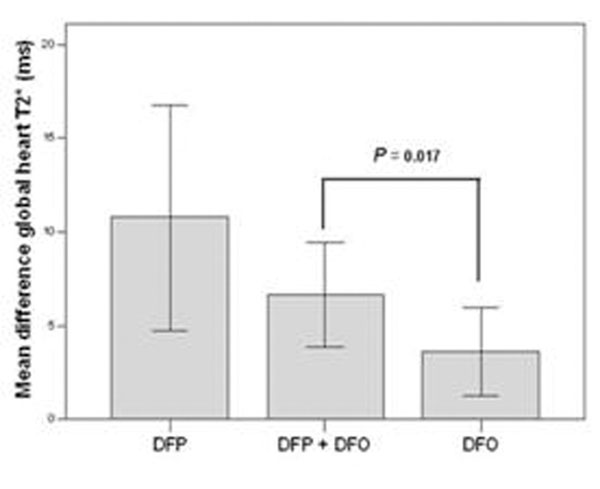


## Conclusions

Prospectively in a large clinical setting over 15 months in TM patients combined therapy DFP+DFO confirmed superior reduction in myocardial iron in comparison to DFO, but no significant differences were found versus DFP monotherapy.

